# Cases Illustrative of the Efficacy of Various Medicines, Administered by Inhalation, in Pulmonary Consumption

**Published:** 1831-04-01

**Authors:** 


CASES ILLUSTRATIVE OF THE EFFICACY OF VARIOUS MEDICINES ADMINISTERED BY INHALATION IN PULMONARY CONSUMPTION, &c. By Sir *Charles Scudamore*, M. D. 8vo. pp. 113. Dec. 1830.

THE total want of success that has attended all specific measures as remedies for real and unequivocal tubercular phthisis, generates a kind of prepossession against every new proposal. The specious, but we think fallacious, argument in favour of the direct application of medicinal substances to the seat of the disease, loses much of its force, when we consider that the excavations left by the softening down of tubercles are not like ulcers on the external surface of the body, which may be cleaned with a sponge or lint, and then dressed with specific ointments. The atmospheric air has very little access to tubercular excavations, which are constantly secreting pus, and for the most part of the 24 hours filled more or less with the same. To expect that iodine can have any effect in healing these excavations, is beyond our faith. Then as to tubercles that have not yet softened down, is it likely that vapour impregnated with iodine and drawn into the air-passages, will have more effect in softening them down than when the medicine is taken into the system by the stomach, and made to permeate the whole vascular system? We much doubt it.

Sir Charles Scudamore has chosen a very unfortunate time, as far as professional feeling is concerned, for bringing forward a system of inhaling for the cure of consumption; since even the Marquesses and Marchionesses are beginning to be ashamed of the quackeries of St. John Long. To mend the matter, Sir Charles has most imprudently chosen to throw a certain degree of mystery about the preparation of iodine to be imployed, and the mode of using it, which has generated a very strong feeling of disapprobation among his professional brethren. The reasons which the author assigns for this procedure, are far from satisfactory. He says that if he unequivocally made known the recipe and the mode of employing it, patients might send to the chymist instead of the physician, and employ it without proper descrimination—by which means discredit might be brought upon a useful remedy, if judiciously applied. But Sir Charles forgets that the same argument might be brought against every remedy in the Pharmacopœia. On this plan the preparations of mercury, their doses, and the modes of employing them in syphilis, hepatitis, and fifty different complaints, ought to have been kept secret, lest non-professional people should tamper with their own health, and employ them unskilfully! In short, the argument is totally untenable; and every physician who means to keep up the respect of his brethren must divest his discoveries and his experiments of every shadow of mystery or concealment. Sir Charles should instantly add an appendix, developing the minutest process employed by him in the inhalation of iodine—though we have very little hope that the remedy will be of any material use in phthisis.

After these remarks, it will not be expected that we should go into a detailed analysis of the work before us. We shall, however, in justice to the author, select two cases, the third and the sixth, which he evidently considers as the least equivocal in favour of iodine inhalation, as a remedy for phthisis, and then our readers may judge for themselves.

"Case III. *Hæmoptysis, succeeded by ulceration; hectic fever, well marked; from all concurrent symptoms the existence of phthisis pulmonalis established; the curative powers of iodine inhalation strongly displayed.*

A female, aged thirty-four, of delicate form, with rather narrow yet not an ill-formed chest, of fair complexion, with dark eyes and white teeth, the mother of several children, having been much debilitated by three miscarriages within the last two years, and suffering from a severe cough, consulted me in February of the present year. In the history of her case, she related that, four years ago, she first contracted a violent catarrhal cough, which had since continued always troublesome, with the exception of an intermission in the summer months; that in January she had coughed up blood to the amount of a teacupful; and from that time had been affected with constant cough, pains of the chest, with quickened and difficult respiration, frequent palpitation of the heart, inability to lie on the right side, and one very distinct paroxysm of hectic fever in the middle of the day, and a slighter one in the evening. There were copious night sweats: she was much wasted in flesh: the catamenia had been suspended two months: the pulse was 120; the animal heat 99°: the expectoration was in quantity about four ounces in the twenty-four hours, of a general puriform appearance, and gave a ring of colours in the optical experiment: the digestive functions were not much disturbed; but the urine deposited much lateritious sediment.

The following indications appeared from the stethoscope and percussion:* the voice was brought distinctly under the tube at the apex of the right lung, and there was obscure *gargouillement* at that part. The sound was dull at the upper part of the right lung, and very remarkably so on percussing the clavicle. The left lung was comparatively in a healthy state.

I prescribed a weak solution of iodine for the inhalation; internally, from one to two minims of the solution of acetate of morphia; and the following draught before rising in the morning:—

℞. Magnes. Sulphat., ʒi.

Infus. Rosæ, ʒxii.

Acidi Hydrocyan. ♏i.

Syrupi Tolutan. ʒi—M. fiat haustus.

The chest all round was washed night and morning with the compound vinegar lotion.

The diet was limited to boiled fish, vegetables, and farinaceous puddings. At the end of a few days she found herself improved, and particularly as to the greater facility of expectorating, more ease of chest, and better respiration. The cough, however, still being very irritable, I added conium to the inhalation.

The mitigation of the symptoms was now very obvious; and, at the end of a fortnight, the amendment was great: but about this period she took cold, and suffered severely for twenty-four hours from disorder of the bowels and from spasms which appeared to proceed from uterine irritation. The cough became more irritable; but otherwise the pulmonary symptoms were not aggravated. I changed the inhaling mixture for one consisting of conium and prussic acid. This indisposition soon yielded to treatment, and the iodine inhalation with conium was resumed, and with an increased proportion of iodine. At the end of a month her appearance was remarkably improved, and all the symptoms were relieved. The pulse was reduced to 80; the animal heat to 95°; the respiration appeared unembarrassed; the cough was comparatively slight; the sputa small in quantity and much improved in character; there was no longer hectic fever; and the night sweats were much lessened. She had gained flesh, and some improvement of strength; yet she still complained of great debility.

She had been most attentive in the use of the inhalation three times a day, and extolled it as the source of her improvement. For the last week she had discontinued the morphia at night, and took no other medicine than the mild aperient draught occasionally. The most urgent symptoms being subdued, I now directed my attention to the improvement of the strength. I prescribed the following draught:—

℞. Acidi Hydrocyan. ♏i.

Decoct. Cinchon. ℥i.

Mist. Amygd. ℥ss.

Aquæ Menth. virid. ʒii.—M. fiat haustus bis die sumendus.

---

* " In this and some of the following cases I had the advantage of an exami-

She was desired to use the inhalation only twice in the day. She took mild animal food each other day, and at dinner two ounces of old port in a tumbler of cold water. She continued the use of the vinegar lotion. She took carriage exercise when the weather was favourable, and walked out occasionally.

In another fortnight I prescribed a saline bark draught, omitting the hydrocyanic acid, and allowed her to take meat or poultry every day. She continued to amend regularly. The catamenia returned. Three months having elapsed, she had recovered so completely that no further treatment appeared to be necessary. For the last week she had inhaled only once a day. She improved in flesh, and was so much stronger, that she declared herself better in health altogether than she had been for six or seven years.

This lady having removed to a distant part of the country, I have no opportunity of ascertaining the present state of her chest by auscultation; but I have the satisfaction of hearing that she continues perfectly well." 25.

Case VI. *Phthisis Pulmonalis; Tubercles in each Lung; great probability of an Ulcer at the apex of the Right Lung; Hectic Fever present; the Iodine inhalation highly beneficial; the Tubercular Irritation removed; and the patient restored to health.*

A gentleman, aged forty-nine, short and slight, and evidently of weak constitution, subject to winter cough, was seized with hæmoptysis some months before the attack of illness which I am about to describe; but the discharge of blood was not large, and did not continue beyond twenty-four hours. He appeared to recover his usual state of health, which was always delicate.

I was consulted in June, and found him affected with very irritable cough, short breathing, a painful state of the chest, with oppression, very disturbed sleep, and night perspirations.

The digestive functions were not materially impaired; yet the appetite was not so good as usual, and the urine deposited lateritious sediment. The bowels were regular, and the liver acted properly.

The expectoration was copious, consistent, of greenish appearance, of faint, disagreeable odour, and it afforded a coloured ring when examined as before described.

He complained of great debility. The pulse was only 60; but I learnt that in health it was of the remarkable slowness of 44 and 46. He had a hectic paroxysm about noon every day. The animal heat was 100. His habits were very temperate; and for many years he had refrained from all fermented liquors.

The following were the indications by the stethoscope and percussion: pectoriloquism at the apex of the right lung, and suspicious at the apex of the left; dull sound in general on the right side, especially at the upper part; dull also at the upper part of the left lung. I drew the conclusion that each lung was tuberculated; that tubercles occupied the right extensively; and that there was, in all probability, some ulceration at its apex.

I directed a blister to the chest; a minim dose of hydrocyanic acid, at noon and at night, in infusion of roses, adding to the day draught some sulphate of magnesia. I prohibited animal food. He entered immediately on the use of the iodine inhalation combined with conium. I enjoined great quiet; for he found himself unfavourably excited by any bodily exercise or by mental exertion.

The chest was much relieved by the blister. On the healing of the skin, I directed the use of the compound vinegar lotion, to be applied just tepid, and afterwards the use of the flesh-brush. He was sensible of a very soothing influence from the inhalation: it caused an easy expectoration, relieved the cough most satisfactorily, and rendered the breathing at once comfortable.

---

nation by Dr. Edwin Harrison, in whose long experience and great tact in auscultation and percussion I have the highest confidence."

In two days, the pulse was 56; the animal heat 98°; the sputa of a creamy white, and still of a faint odour. The tongue was coated with whitish flakes, and the gums were spongy, as if from mercury: but there was no ptyalism. This state of the tongue and gums was in part produced by the inhalation; an effect, particularly as regards the tongue, which I have occasionally witnessed. But these effects either pass away, or become too slight to be regarded, as the patient becomes accustomed to the use of the inhalation.

As there appeared to be yet too much excitement in the system, I increased the dose of hydrocyanic acid to three minims twice a day; but I did not continue these proportions more than three days; for, as the symptoms abated, I resumed the dose of one minim.

The whole plan of treatment agreed perfectly. In a fortnight, the state of the patient was surprisingly ameliorated; and the appetite was much improved. I allowed him to eat boiled fish or mild animal food, on alternate days.

At the end of three weeks, the amendment was still more confirmed. The pulse was reduced to its natural standard of 44; the animal heat to 97°; the respiration was quite comfortable; the cough very slight; the sputa small in quantity and consisting chiefly of frothy mucus; the nights were passed with good sleep and freedom from perspiration; the tongue was almost clean, and the gums nearly restored to their natural state, although the inhalation had been regularly continued three times a day; the urine was clear.

I never witnessed in so short a time such a happy change in the looks as this gentleman displayed. The hectic flush of the cheek had passed away; there was a cheerful expression of countenance; and there was some recovery of flesh. He spoke in the highest terms of praise of the inhalation; and, as this gentleman was very intelligent, and minute in his observations, I attached the more importance to his report. He stated, that it invariably gave ease and comfort to his chest; quickly improving the breathing, and relieving the cough.

The circulation being now free from excitement, and the opportunity therefore presenting itself for the adoption of more restorative means, I prescribed the infusion of the cortical part of sarsaparilla in lime water, with the addition of the strong syrup of the cortical part, and Brandish's alkaline liquor, to be taken twice a day, in admixture with an equal portion of hot milk.

The patient continued to improve progressively in the most favourable manner. Not one untoward circumstance occurred. He reduced the use of the inhalation to twice a day, in three weeks from the commencement; in five weeks, to once; and discontinued it wholly at the end of two months. At this period he was free from all symptoms of illness; quite relieved from cough, with recovered flesh and strength; the pulse at its natural standard of 44; the animal heat 96°; the appetite, the digestive functions, and the sleep, all natural. At the present time (October,) he continues to find himself in much better health than he has been for two years past. His breathing is so greatly improved that he can walk six or seven miles a day without inconvenience." 48.

We have now exhibited two of the best cases in the book, and, at an earlier period of life we should have been inclined to cry out EUREKA! Recollecting, however, the host of cases, still more decidedly phthisical than the above, which were cured at Bristol, by Dr. Beddoes, some twenty or more years ago, and by a somewhat similar process—remembering that every year, since the commencement of our practice, some infallible panacea or mode of treatment has been proclaimed for a disease that is almost invariably fatal, we have read the above, and all the other cases narrated by Sir Charles, without being elated, and without being convinced. The inhalation of soothing and medicinal vapours may prove useful, in various affections of the air-tubes and their lining membrane, and the publication before us will, no doubt, excite ardent hopes in the hectic breast. So far it will be very beneficial, in a pecuniary point of view, to the author; but we doubt much, whether it will add permanently to his fame, in the records of medicine.

